# UVB-induced DHODH upregulation, which is driven by STAT3, is a promising target for chemoprevention and combination therapy of photocarcinogenesis

**DOI:** 10.1038/s41389-019-0161-z

**Published:** 2019-09-24

**Authors:** Mohsen Hosseini, Léa Dousset, Pauline Michon, Walid Mahfouf, Elodie Muzotte, Vanessa Bergeron, Doriane Bortolotto, Rodrigue Rossignol, François Moisan, Alain Taieb, Anne-Karine Bouzier-Sore, Hamid R. Rezvani

**Affiliations:** 1 Univ. Bordeaux, INSERM, BMGIC, UMR 1035, F-33076 Bordeaux, France; 2 Univ. Bordeaux, INSERM, MRGM, U1211, F-33076 Bordeaux, France; 3 Centre de Référence pour les Maladies Rares de la Peau, CHU de Bordeaux, Bordeaux, France; 4 Service de Dermatologie Adulte et Pédiatrique, CHU de Bordeaux, Bordeaux, France; 5 Centre de Résonance Magnétique des Systèmes Biologiques, CNRS-Université Bordeaux UMR, 5536 Bordeaux, France

**Keywords:** Squamous cell carcinoma, Nucleotide excision repair, Chemotherapy

## Abstract

The leading cause of cutaneous squamous cell carcinomas (cSCCs) is exposure to ultraviolet radiation (UV). Unlike most other cancers, the incidence rates of cSCCs are still on the rise and the treatment options currently available are limited. We have recently found that dihydroorotate dehydrogenase (DHODH), which is the rate-limiting enzyme in the de novo pyrimidine synthesis pathway, plays a critical role in UVB-induced energy metabolism reprogramming. Using a multistage model of UVB radiation-induced skin cancer, we show that UVB-induced DHODH upregulation is mainly regulated transcriptionally by STAT3. Our results indicate that chronic inhibition of DHODH by leflunomide (LFN) blocks UVB-induced tumor initiation. Human tumor xenograft studies showed that LFN treatment reduces growth of established tumors when used in combination with a genotoxic agent, 5-fluorouracil (5-FU). Our data suggest that DHODH is a promising target for chemoprevention and combination therapy of UVB-induced cSCCs.

## Introduction

Exposure to ultraviolet (UV) radiation from the sun is the most significant risk factor resulting in non-melanoma skin cancers (NMSCs), including basal cell carcinomas and cutaneous squamous cell carcinomas (cSCCs), the most common types of human malignancies worldwide. An estimated 5.4 million cases of NMSCs were affecting 3.3 million patients among the US population in 2012^1^. The rate of NMSCs is increasing by 5–7% per year, mainly due to UV exposure and population ageing^2^. cSCCs typically manifest as a spectrum of progressively advanced malignancies, ranging from precursor actinic keratosis (AK) to in situ, invasive, and finally metastatic cSCCs^3^. A recent US study estimated that 3900–9000 patients died from cSCCs in 2012^4^. Thus, it is crucial to preserve the general high chances of cure of cSCCs by a careful evaluation and proper early management of all cases, and not underestimate the potential aggressiveness of this tumor^5,6^.

The most commonly used therapies to treat AKs, such as cryotherapy, laser or FDA-approved topical 5-fluorouracil (5-FU) or imiquimod^7^, have significant painful side-effects and a high level of post-treatment recurrence. For cSCCs, surgery is the curative treatment, followed by radiotherapy and chemotherapy, which are mainly used as adjuvant or palliative treatment in advanced cSCCs^7^. The most commonly used chemotherapy agents for cSCCs in stage III and IV with nodal or distant metastasis are 5-FU/cisplatin, 5-FU/carboplatin, and paclitaxel/carboplatin combinations^8^. Targeted therapies such as epidermal growth factor receptor (EGFR) inhibitors have shown efficacy but no sustained remission. More recently, immunotherapy and checkpoint inhibitors showed promising results^9^. Overall, these data suggests the urgent necessity to improve the level of prevention and protection for skin cancer and the evaluation of new therapeutic strategies.

Leflunomide (LFN), an FDA-approved drug for the treatment of rheumatoid arthritis, is an inhibitor of the enzyme dihydroorotate dehydrogenase (DHODH)^10–12^. As the rate-limiting enzyme in the de novo pyrimidine synthesis pathway, DHODH catalyzes the conversion of dihydroorotate to orotate in the fourth step of the six enzymatic reactions of this pathway. Inhibition of DHODH prevents the synthesis of pyrimidines and consequently impairs the synthesis of pyrimidine derivatives, such as the nucleotide bases cytosine and thymine. We have recently shown that this enzyme, which is located in the inner mitochondrial membrane, plays a critical role in UVB-induced energy metabolism reprogramming^13^. Indeed, upregulation of DHODH was shown to be important in maintaining higher electron transport chain (ETC) activity in irradiated skin and in ensuring the coordination of ATP generation and persistent nucleotide biosynthesis. The latter is indeed necessary for the repair of damaged DNA, which is one of the major deleterious effects of exposure to UVB^13^. The most frequent lesions arising from UVB exposure are cyclobutane pyrimidine dimers (CPDs) and pyrimidine (6–4) pyrimidone photoproducts (6-4PPs). They are mainly repaired by the nucleotide excision DNA repair (NER) pathway whose action results in the release of a 24-mer to 32-mer oligonucleotide comprising the damaged base(s) and its replacement by a newly synthesized DNA^14^.

In this study, we show that UVB-induced DHODH upregulation is mainly regulated transcriptionally by STAT3. Furthermore, we show that inhibition of DHODH by LFN blocks UVB-induced tumorigenic transformation of keratinocytes. Given the importance of DHODH for nucleotide biosynthesis, we finally tested the efficiency of LFN for the therapy of cSCC. Results indicate that the growth of established A431 and SCC-15 cell xenografts is significantly reduced when LFN is used in combination with a genotoxic agent. Our data demonstrate the potential of DHODH as a novel target for the prevention and combination therapy of UVB-induced cSCCs.

## Results

### UVB irradiation results in DHODH upregulation

**Fig. 1:**
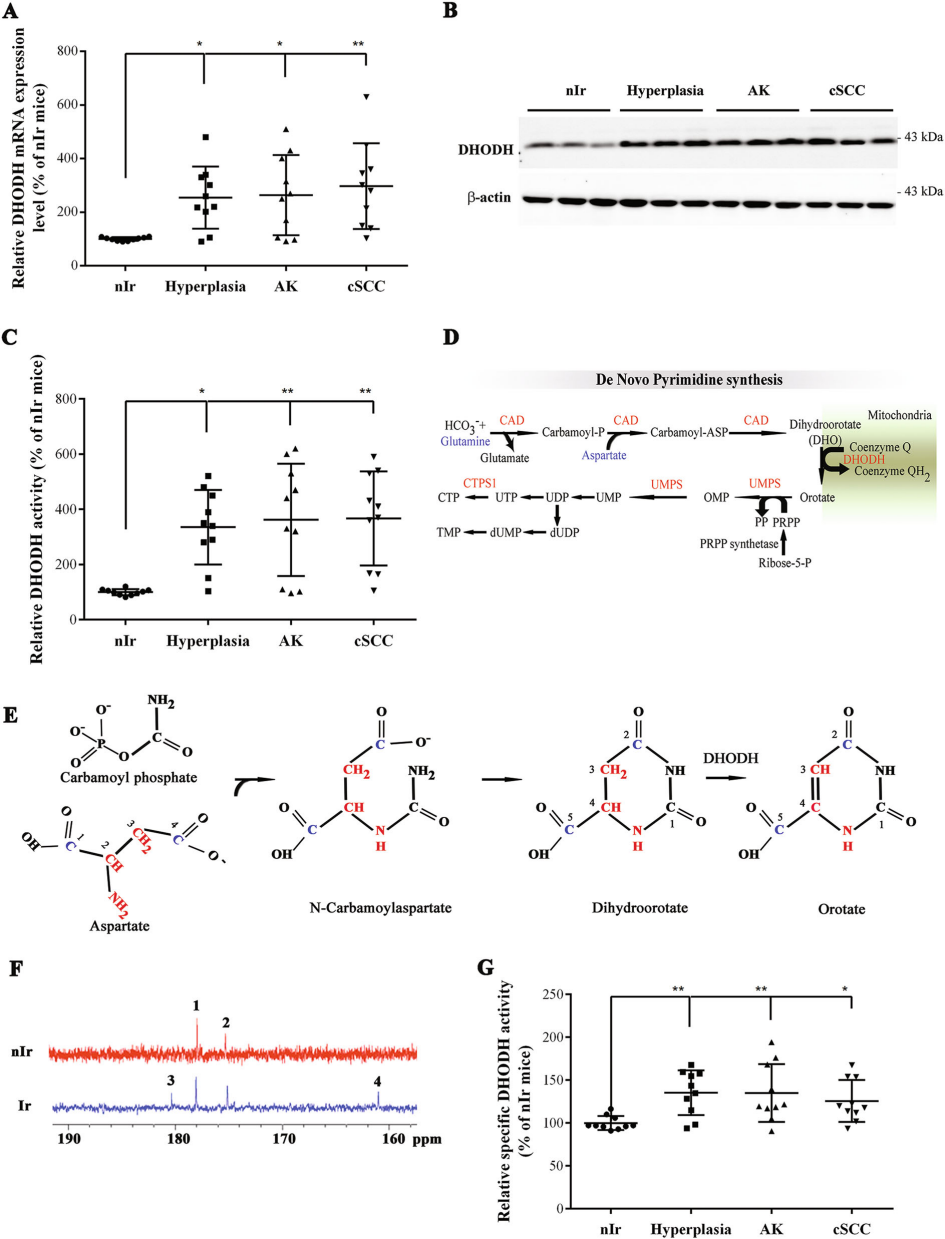
Chronic UVB exposure results in DHODH overactivation SKH-1 mice exposed to chronic UVB irradiation. **a** The relative levels of DHODH mRNA were quantified by quantitative reverse transcription PCR. **b** Total protein extracts of skin biopsies at different stages of tumorigenesis were assessed for expression of DHODH by western blot. β-actin was used as a loading control. Full-length blots are presented in Fig. S1. **c** DHODH activity was measured in skin biopsies at different stages of UVB-induced carcinogenesis. **d** DHODH is the only mitochondrial enzyme of the de novo pyrimidine synthesis pathway that converts dihydroorotate to orotate. **e**, **f** Cells were incubated for 4 h with [1,4-^13^C] aspartate. **e** Schematic depicting atoms of orotate that are derived from aspartate and carbamoyl phosphate. Blue color represents labeled carbons from aspartate, red color represents non-labeled carbons and nitrogen from aspartate, and black color represents carbon and nitrogen derived from carbamoyl phosphate. **f** ^13^C-NMR spectra of perchloric acid cell extract after 4 h of incubation with [1,4-^13^C] aspartate. nIr non-irradiated cell, Ir irradiated cell. 1: Aspartate C4, 2: Aspartate C1, 3: orotate C2, and 4: orotate C5. **g** Specific DHODH activity was measured by normalization of DHODH activity to its expression level in each sample. *N* = 10 [(A–C, G)] and 6 [(F)] mice per group *P < 0.05 and **P <0.01 for irradiated versus non-irradiated mice. PRPP phophoribosylpyrophosphate, CAD carbamoyl-phosphate synthetase 2, aspartate transcarbamylase, and dihydroorotase, CTPS1 CTP synthase, DHODH DHO dehydrogenase, OMP orotidine 5′-monophosphate, UMPS uridine 5′-monophosphate synthase

We have recently shown that UVB-induced skin tumorigenesis is associated with altered metabolism^13^ and that DHODH fuels mitochondrial respiration to coordinate DNA repair and ATP synthesis. To investigate the effects of chronic UVB irradiation on DHODH expression, SKH-1 hairless mice which closely mimic photocarcinogenesis in humans^13^ were irradiated three times a week with 150 mJ/cm^2^ UVB. We measured the expression and the activity of DHODH in mouse skin at different stages of UVB-induced carcinogenesis (Fig. 1a–c). DHODH mRNA (Fig. 1a) and protein expression (Fig. 1b) levels as well as its enzyme activity (Fig. 1c) were markedly upregulated at a very early phase of UVB-induced tumorigenesis and this up-regulation persisted during the subsequent steps of carcinogenesis. As already mentioned, DHODH catalyzes the conversion of dihydroorotate to orotate in the fourth step of pyrimidine synthesis, in which glutamine and aspartate are used as precursors (Fig. 1d). Functional activation of this pathway was then verified by tracing analysis. To this end, epidermal cells isolated from non-irradiated and irradiated mice were incubated for 5 h in the medium supplemented with labeled [1,4-^13^C] aspartate. Results indicated that orotate, a central intermediate of pyrimidine synthesis, became labeled on carbons 2 and 5 only in irradiated cells (Fig. 1e). Given that specific DHODH activity was only about 1.5-fold higher in irradiated skin than in non-irradiated skin (Fig. 1f), we concluded that DHODH was mainly regulated transcriptionally in irradiated skin.

### UVB-induced transcriptional upregulation of DHODH is driven by STAT3

**Fig. 2:**
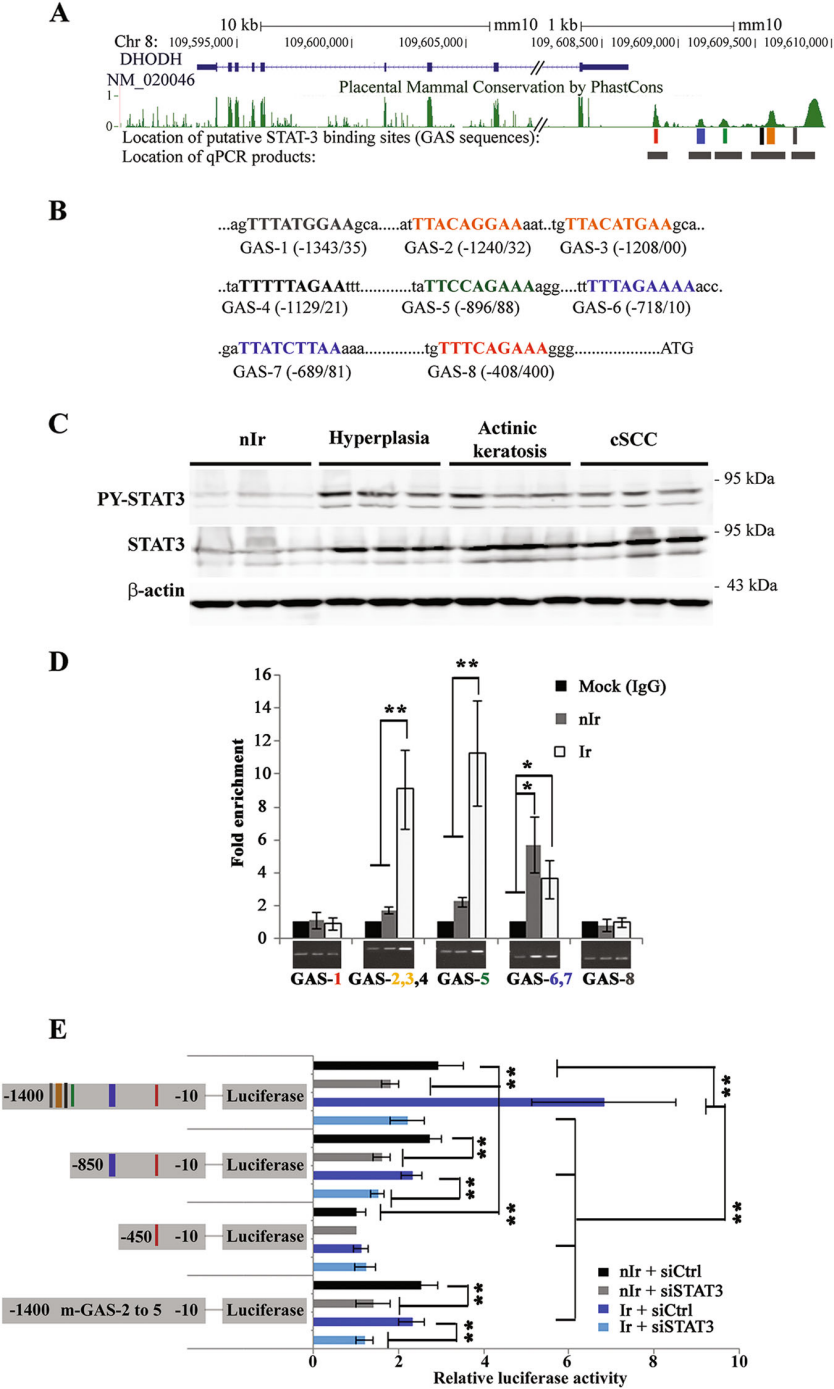
UVB-induced DHODH overexpression is regulated via a STAT3-dependent mechanism. **a** Representation of the DHODH gene and its promoter in the UCSC genome browser, including location of the eight motifs matching the consensus STAT3-binding site [TTNNNNNAA)] and the location of PCR products for ChIP-qPCR. **b** Eight nucleotide-sequences matching the consensus STAT3-binding site (GAS-1–GAS-8) in the upstream region of mouse DHODH gene are numbered in relation to the translational start codon, ATG. **c** The expression of STAT3 was assessed in mouse skin specimens at different stages of tumorigenesis. Upregulation of STAT3 expression persisted at different stages of UVB-induced carcinogenesis. Full-length blots are presented in Fig. S2. **d** Irradiated and non-irradiated skin were subjected to ChIP assay using an anti-STAT3 antibody. Bands indicate PCR products using primers that span the indicated GASs. The relative levels of corresponding precipitated GAS fragments following ChIP were quantified by qRT-PCR. **e** Luciferase reporter plasmids containing indicated fragments were transfected into siCtrl-transfected or siSTAT3-transfected keratinocytes isolated from irradiated or non-irradiated skin. *N* = 6 mice per group. *P < 0.05 and **P < 0.01

To establish what accounts for *DHODH* gene overexpression following irradiation, software analysis of the promoter region of *DHODH* gene was performed. Results revealed that eight putative interferon-gamma-activated sequences (GAS), with the general consensus sequence of TTNNNNNAA^15^, are located in the 1.4 kb region upstream from the ATG translation initiation codon (Fig. 2a, b). Of note, the protein family that can bind to GAS sequences is the signal transducer and activator of transcription (STAT). Among the members of this family, STAT3 has been shown to play a critical role during UVB-induced carcinogenesis. Indeed, *Stat3*-deficient mice were resistant to UVB-induced skin carcinogenesis and, inversely, the formation of skin tumors was accelerated in mice overexpressing *STAT3*^16^. These data prompted us to examine whether STAT3 may account for *DHODH* overexpression in irradiated skin by direct binding to the promoter region of *DHODH*. To examine this hypothesis, we first verified the expression of STAT3 and its phosphorylated form at tyrosine-705 (pY-STAT3) at different stages of carcinogenesis. STAT3 and pY-STAT3 were up-regulated at a very early phase of UVB-induced tumorigenesis and this up-regulation persisted at different stages of carcinogenesis (Fig. 2c). We then performed ChIP experiments with the primers spanning eight putative GAS (Fig. 2a, d). While the region containing GAS-6 and 7 immunoprecipitated in both non-irradiated and irradiated skin, immunoprecipitation of GAS-2 to GAS-5 was substantially increased in UVB-irradiated skin. Luciferase reporter plasmid, in which the 1.4 kb region upstream from the ATG translation initiation codon of *DHODH* had been cloned upstream of luciferase, was then used to further characterize the role of GASs in the regulation of *DHODH* expression following UVB-irradiation. This construct was transiently transfected into epidermal cells isolated from non-irradiated and irradiated mice. Results revealed that the basal activity of this promoter in irradiated cells was higher than that of non-irradiated cells (Fig. 2e). STAT3 downregulation or truncating the promoter to −450 bp led to a significant decrease in basal activity of this promoter and totally abrogated the chronic UVB irradiation-induced upregulation of the luciferase activity (Fig. 2e). Of note, to rule out “off target” effects of siSTAT3, these experiments were performed with two distinct siRNAs against STAT3. Since both siRNAs had similar effects, only the results of one of them have been shown in this figure. Moreover, substituting two T nucleotides in the GAS-2, 3, 4, and 5 of the luciferase reporter plasmid for two G nucleotides totally abrogated the chronic UVB irradiation-induced upregulation of the luciferase activity (Fig. 2e). Overall, our results show that STAT3 plays a positive role both in basal and chronic UVB-induced DHODH expression.

### Inhibition of DHODH activity blocks UVB-induced tumor formation

**Fig. 3:**
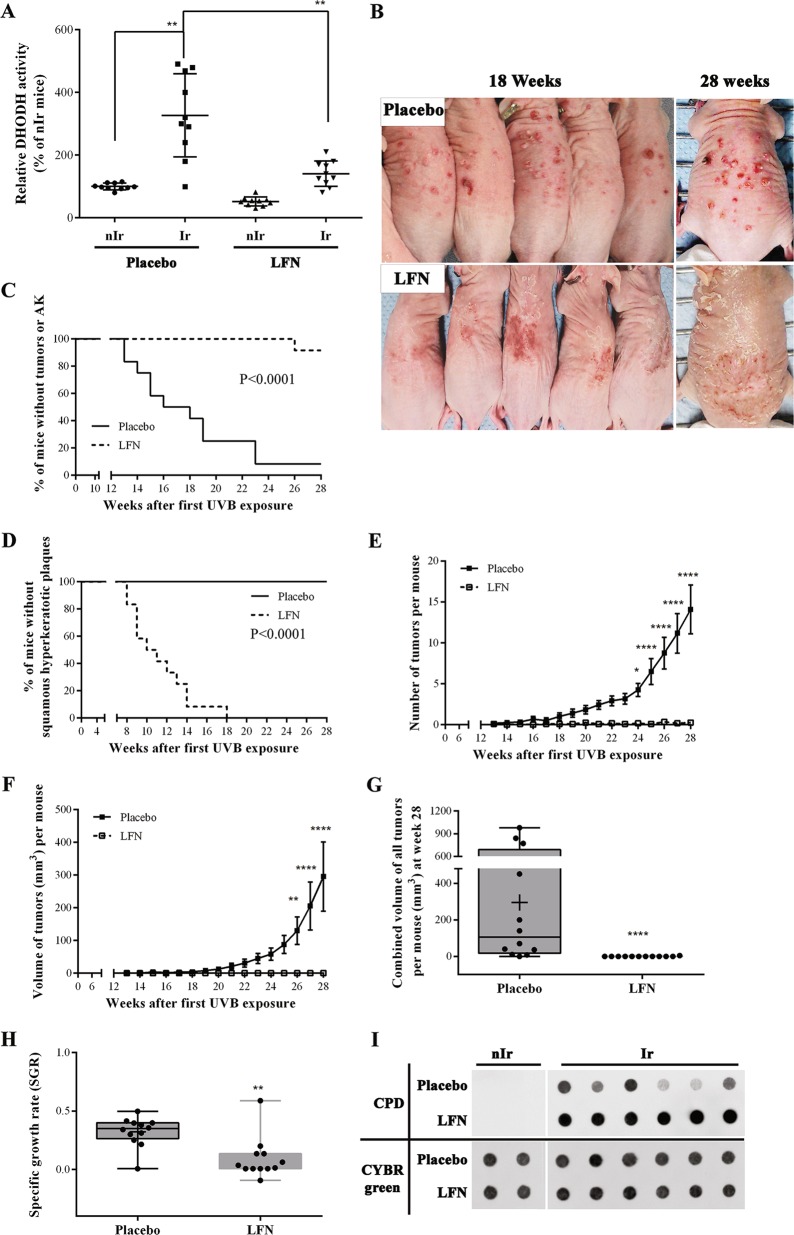
Inhibition of DHODH using LFN treatment results in hypersensitivity to UVB exposure. One-month-old SKH-1 mice were subjected to chronic UVB irradiation ± intraperitoneal injection of either LFN (20 mg/kg/day) or placebo. **a** The relative DHODH activity in irradiated and non-irradiated skin was measured at 8 weeks after irradiation. Increased DHODH activity in UVB-irradiated skin samples was blocked following treatment of mice with LFN. **b** Photographs are representative examples of placebo-treated and LFN-treated mice after 18 and 28 weeks of chronic UVB irradiation. **c**, **d** The percentage of tumor-free mice **c** and desquamative feature-free mice **d** were assessed at indicated times. **e**, **f** the numbers **e**, and the combined volumes **f** of tumors per mouse were recorded at different intervals. **g** The distribution of the mean volume of all single tumors/mouse is presented at week 28 of chronic UVB irradiation. **h** The specific tumor growth rate SGR was estimated for each mouse according to the following equation: SGR = Ln2/DT, in which DT = doubling time. **i** Cyclopyrimidine dimer (CPD) levels were quantified in mouse skin at 8 weeks of chronic UVB irradiation by immuno-dot blot analysis. Full-length blots are presented in Fig. S3. *N* = 12 [(A–I)] mice per group *P < 0.05, **P < 0.01, and ****P < 0.0001

To investigate the impact of DHODH upregulation on the susceptibility to UVB-induced skin cancer, DHODH activity was inhibited using LFN, a non-specific inhibitor of DHODH and an FDA-approved drug for the treatment of rheumatoid arthritis. LFN (20 mg/kg/day) was administered intraperitoneally each day and 1 h before each UVB exposure. In the absence of UVB exposure, neither placebo nor the LFN treatment showed any adverse effect on skin (data not shown). Assessment of DHODH activity at 8 weeks after irradiation indicated its efficient inhibition in LFN-treated mice (Fig. 3a). Monitoring placebo-treated and LFN-treated mice during chronic UVB irradiation indicated that the latter failed to develop malignant lesions (Fig. 3b–d). Indeed, while none of the placebo-treated mice had any obvious abnormalities up to week 12 of irradiation, 7 out of the 12 mice exhibited at least one AK lesion at 18 weeks (Fig. 3c–e). More than 90% of these mice had an average of 14 tumors of variable size, exhibiting an exponential increase in size at 28 weeks of irradiation (Fig. 3e–h). In contrast, 8 out of the 12 LFN-treated mice developed moderate squamous hyperkeratotic plaques 12 weeks after UVB irradiation (Fig. 3b–d). Eighteen weeks after chronic UVB irradiation, all these mice presented desquamative features with a hyperkeratotic epidermis (Fig. 3b–d). However, only 1 of these 12 mice displayed three keratotic tumors up to 28 weeks after irradiation (Fig. 3c, g).

### Pyrimidine supplementation restores UVB-induced tumorigenic transformation of keratinocytes in LFN-treated mice

Since DHODH is a critical enzyme in the pyrimidine de novo biosynthesis pathway, we next sought to investigate whether the DHODH inhibition-mediated reduction in pyrimidine synthesis and subsequent decreased DNA repair efficiency could explain the absence of tumor formation in LFN-treated mice despite their UVB-hypersensitive phenotype. To test this hypothesis, we first assessed the repair capacity of placebo-treated and LFN-treated mice irradiated for 12 weeks by quantifying the levels of CPDs in the whole skin (Fig. 3i). Results obtained by immuno-dot blot indicated a substantial increase in the CPD level in epidermal DNA of LFN-treated mice compared to control counterparts.

**Fig. 4:**
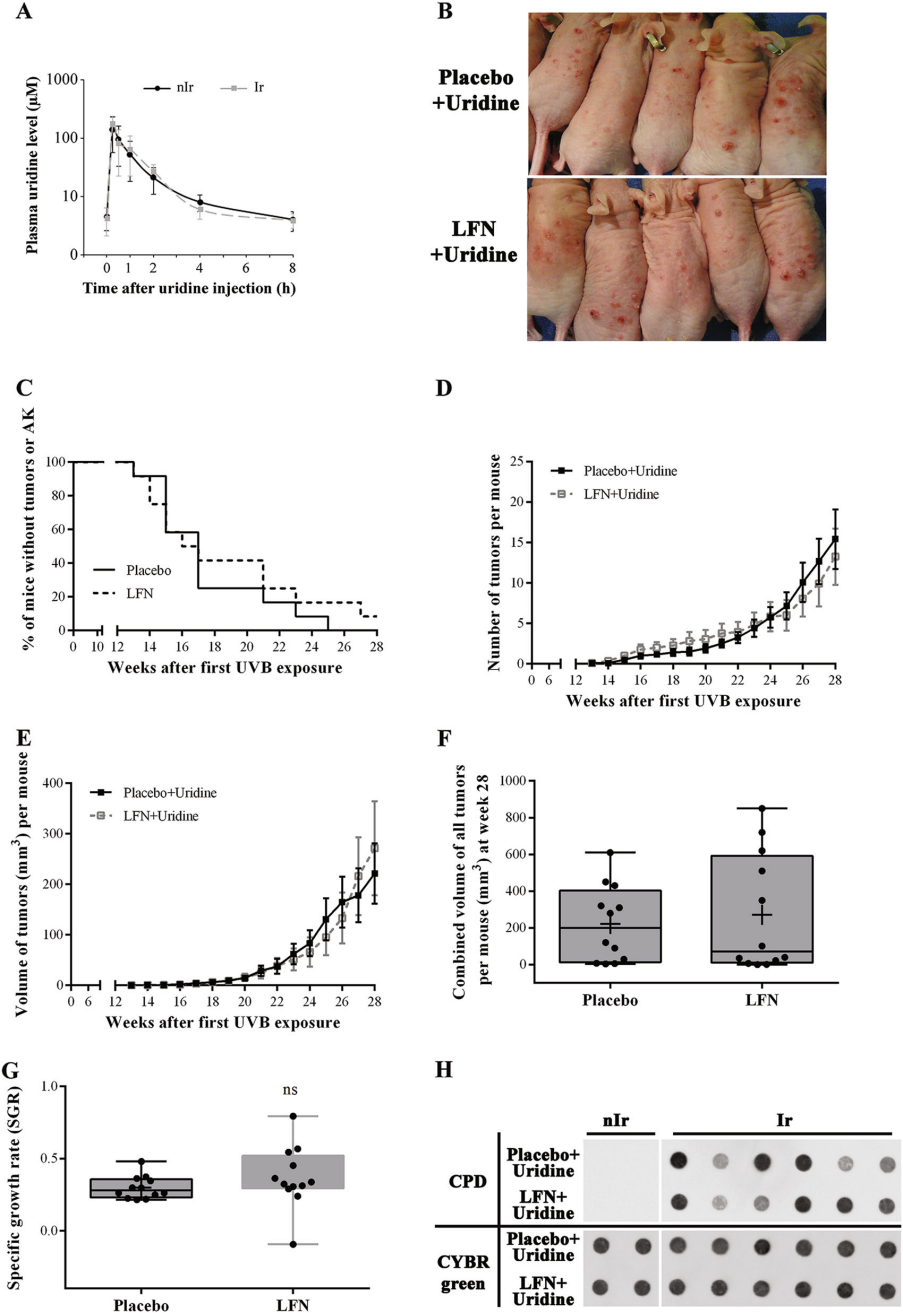
Uridine supplementation largely restored DHODH inhibition-mediated decreased DNA repair capacity and hypersensitivity to UVB. One-month-old SKH-1 mice were subjected to chronic UVB irradiation + intraperitoneal injection of either LFN (20 mg/kg/day) or placebo + intraperitoneal uridine. **a** Time courses of plasma uridine concentrations after an i.p. injection of exogenous uridine at week 8 of chronic UVB exposure. **b** Photographs are representative examples of mice treated with either placebo + uridine or LFN + uridine after 18 weeks of chronic UVB irradiation. **c–e** The percentage of tumor-free mice **c**, the tumor numbers **d**, and the volume **e** of tumors per mouse were assessed at indicated times. **f** The distribution of the mean volume of all single tumors/mouse is presented at week 28 of chronic UVB irradiation. **g** The specific tumor growth rate SRG was estimated for each mouse. **h** Immuno-dot blot analysis reveals the same quantity of cyclopyrimidine dimer (CPD) in both LFN-treated and placebo-treated mice supplemented with uridine upon chronic UVB irradiation. Full-length blots are presented in Fig. S3. *N* = 12[(A–H)] mice per group

To test functionally whether the observed phenotype in LFN-treated mice was due to the decreased pyrimidine biosynthesis, supplementation with uridine (i.p. injection of 100 mg/kg/day uridine) was tested. Of note, uridine levels in the plasma peaked rapidly after each injection and returned to pretreatment levels within 8 h (Fig. 4a). Monitoring the mice during chronic UVB irradiation indicated that supplementation with uridine blocked the formation of hyperkeratotic plaques (Fig. 4b). Indeed, assessment of the incidence, number, volume, and growth rate of tumors revealed no significant difference between the group receiving LFN and uridine and mice treated with uridine alone (Fig. 4c–g). Furthermore, there was no significant difference between the two groups concerning the efficiency to remove the UV photoproduct (Fig. 4h).

Altogether, these results showed that uridine supplementation restored UVB-induced tumor formation in LFN-treated mice and blocked formation of hyperkeratotic plaques in them.

### Synergistic anti-tumoral effect of LFN and 5-FU on established human tumors

**Fig. 5:**
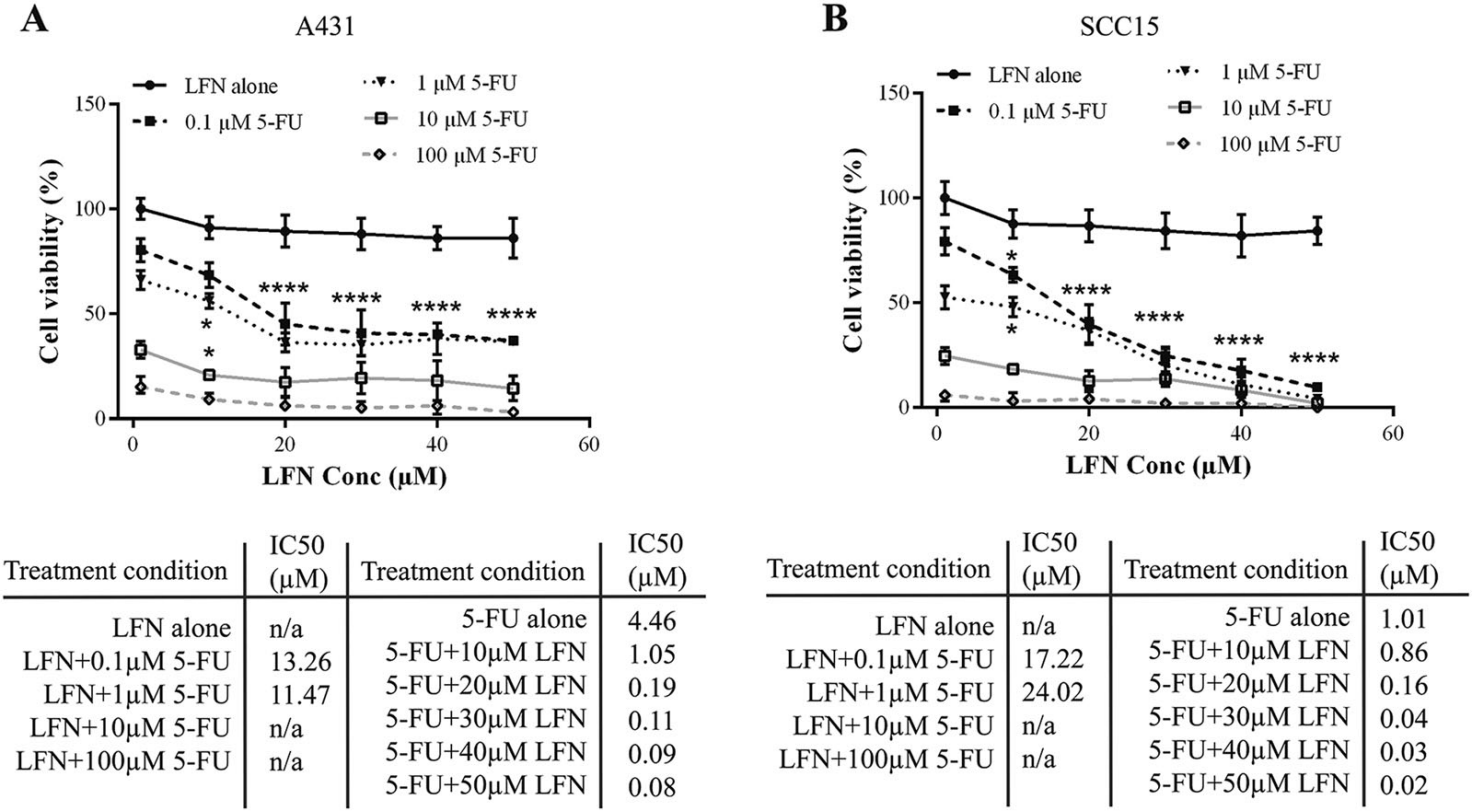
The combination of LFN and 5-FU reduces tumor growth in vivo. **a**, **b** The effect of different concentrations of LFN and 5-FU on the viabilities of A431 **a** and SCC-15 **b** was measured at 24 h after treatment. The statistical analysis on the graphs compares the drug combinations to each respective treatment alone. All values are represented as a percentage (%) relative to the placebo. Data is presented as the mean ± SEM of three independent experiments each performed in triplicate

We then sought whether LFN could slow the progression of human SCC. To this end, we first characterized the effect of LFN on the viability of A431 and SCC-15 cell lines. As a control, we used the genotoxic anti-cancer drug 5-flurouracil (5-FU). Neither A431 nor SCC-15 were sensitive to LFN (Fig. 5a, b). However, when LFN was used in combination with 5-FU, a remarkable synergistic effect was detected (Fig. 5a, b). The most statistically significant combination of LFN and 5-FU was 50 μM LFN and 1 μM 5-FU, i.e. a ratio of 50:1.

**Fig. 6:**
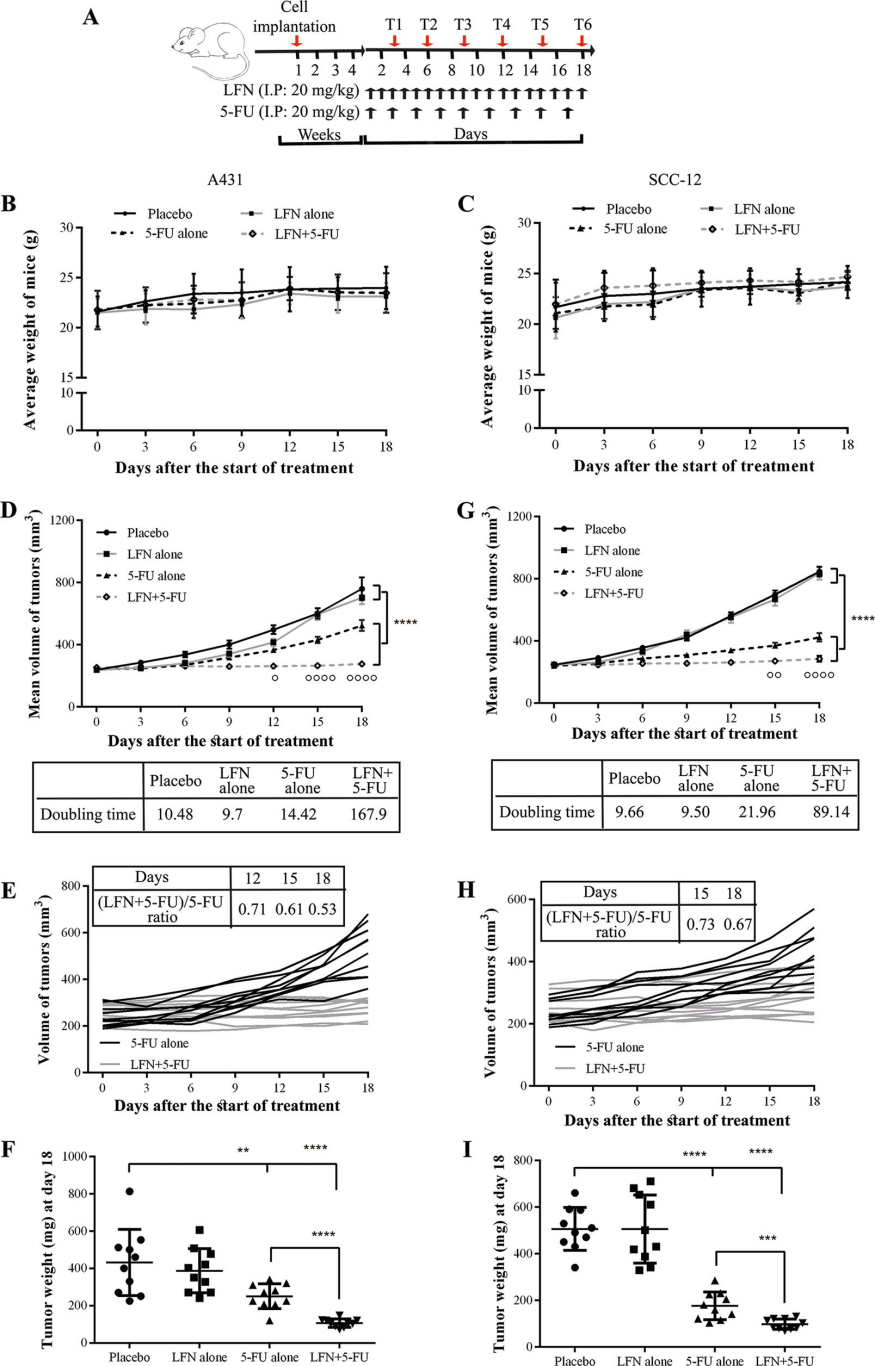
The combination of LFN and 5-FU reduces tumor growth in vivo. **a** A431 and SCC-15 cell (5 × 10^5^) were injected subcutaneously into NSG mice. When tumors were palpable (4-weeks post-xenografting), mice were treated daily with placebo, LFN, 5-FU, or combined 5-FU and LFN for 18 days. Tumor volumes were measured at six time points (T1–T6). **b**, **c** The body weight of mice after treatment was measured at the indicated time points. Results represent the mean ± SD. **d** The combination of LFN and 5-FU reduced the average A431 tumor volume greater than either drug alone. Data is presented as the mean ± SEM of one independent experiment. Statistical analysis (two-way ANOVA with Bonferroni’s post-hoc test) compares the combination therapy versus each drug alone. ****P ≤ 0.0001. °*P* ≤ 0.05, °°°°*P* ≤ 0.0001 for combined 5-FU and LFN vs. 5-FU. **e** Tumor growth curves of individual A431 tumors in mice treated with 5-FU (black lines) and 5-FU plus LFN (gray lines). The effect of each treatment on mean volume of tumors at indicated day are shown at the top of the panel. **f** The combination of leflunomide and 5-FU reduced tumor weight greater than either drug alone. **g–i** The mean volume of tumors at indicated time points **g**, individual tumor growth curves **h**, and the weight of tumors at day 18 **i** are shown for transplanted SCC-15 cells. *N* = 10 mice per group, one injection per mice. *P < 0.05, **P ≤ 0.01, ***P ≤ 0.001, ****P ≤ 0.0001

To test the effect of LFN on SCC growth in vivo, we transplanted both cell lines into immunocompromised mice. After 4 weeks of tumor growth, mice were divided into four groups receiving either placebo alone, LFN alone, 5-FU alone, or combined LFN and 5-FU (10 animals/group) (Fig. 6a). As shown in Fig. 6b, c, body weight loss was not observed in any treatment group. Moreover, any difference in mouse behavior among the different groups was noted, suggesting that all treatments were well-tolerated. In the placebo group, the average A431 tumor volume increased from 239 mm^3^ on day 0–760 mm^3^ on day 18, indicating a steady increase in tumor growth over the course of the experiment (Fig. 6d). LFN treatment alone did not reduce tumor volume when compared to placebo-treated tumors. In contrast, 5-FU treatment significantly reduced the average tumor volume, even though the tumors continued to grow during the course of the experiment. However, when combined LFN and 5-FU was administered, tumor growth was suppressed, with tumor volumes remaining steady at the same size over the 18-day treatment period (Fig. 6d, e). When weighed at the end of experiment, tumors weighed significantly less in mice treated with combined LFN and 5-FU than in the other groups (Fig. 6f). Similar results were obtained when SCC-15 were transplanted into NSG mice and treated with placebo, LFN, 5-FU, and 5-FU plus LFN (Fig. 6g–i), even though these tumors were more sensitive to 5-FU than the A431 tumors.

Taken together, our data show that the combination of LFN and 5-FU prevents tumor growth in vivo.

## Discussion

We found that DHODH was upregulated at a very early phase of UVB-induced carcinogenesis and its inhibition blocked the tumorigenic transformation of damaged keratinocytes. Indeed, despite manifesting hyperkeratosis, mice treated with an inhibitor of DHODH developed neither actinic keratosis nor skin tumors following exposure to chronic UVB irradiation.

There is now growing evidence showing that several branches of metabolism are affected during malignant transformation to support both the energy demands of cancer cells and their anaplerotic fluxes, which that are necessary for providing cellular building blocks, such as nucleic acids, proteins, and membranes^17–19^. We have already shown that reprogramming of the energy metabolism occurs at a very early phase of UVB-induced carcinogenesis and that mice harboring an impaired ETC fail to develop premalignant and malignant lesions owing to their decreased DNA repair capacity and increased apoptotic cell death^13^. We now show that inhibition of the mitochondrial DHODH enzyme, which couples the de novo pyrimidine biosynthesis pathway to ETC fluxes, results in decreased DNA repair capacity and prevents tumor formation in mice chronically exposed to UVB. The appearance of tumors and the blocking of hyperkeratotic plaque development in LFN-treated mice upon uridine supplementation confirms the importance of nucleotide biosynthesis in determining the fate of cells upon exposure to genotoxic stressors such as UV irradiation. These results suggest that the importance of DHODH in malignant transformation of a cell is dependent on cellular demands for nucleotide biosynthesis and that DHODH plays a key role in malignant transformation of a cell under chronic genotoxic stress.

Increased intracellular pyrimidine nucleotide levels have also been reported when cells are exposed to other genotoxic stressors such as chemotherapy agents^20^. Therefore, the pyrimidine biosynthesis pathway is a promising metabolic target for enhancing the efficacy of chemotherapy and limiting the emergence of resistance. In support of this notion, the combination of doxorubicin and LFN, as well as the combination of MEK inhibitor and LFN have recently been proposed as a promising combination therapy for breast cancer^20^ and melanoma^21^, respectively. In line with these data, our results show that if A431 xenografted tumors are subjected to LFN in combination with a genotoxic agent such as 5-FU, a significant synergistic anti-tumor effect occurs (Fig. 6). Considering the high cytotoxicity of chemotherapy agents especially in elderly patients, this combination therapy could reasonably be expected to be efficient.

Although several drugs target DHODH^22–25^, it has received little attention as a therapeutic target for cancer. As the rate-limiting enzyme in pyrimidine biosynthesis, DHODH is now becoming an attractive target for anti-cancer therapy. DHODH inhibitors have been shown to inhibit the growth of a wide variety of human solid tumors both in vitro and in vivo^21,26–29^. Moreover, it has been recently shown that cancer cells without mitochondrial DNA (mtDNA) do not form tumors unless they import functional mtDNA from host stroma cells. The key step in this event is reconstitution of DHODH-driven pyrimidine biosynthesis^30^. Several studies have also highlighted the significant anti-tumoral effect of DHODH inhibitors on patient-derived tumors^31–34^. The effects of LFN and other DHODH inhibitors on tumor growth, such as brequinar sodium and 4SC-101 have been ascribed to their ability to affect the expression of cell cycle regulators, such as cyclin D2 and pRb expression, the phosphorylation of some proteins, such as protein kinase B, p70S6K, and eukaryotic translation initiation factor 4E-binding protein-1, and/or cellular energy metabolism^13,25,27,35^. Moreover, activation of p53 and p53-dependent apoptosis as a downstream effect of DHODH inhibition-mediated depletion of pyrimidines has been proposed as the fourth mechanism^36^. A recent study showed that there is a strong link between expression of DHODH, tumor growth rate and sensitivity to DHODH inhibitors^35^. Along this line, it has been shown that relatively slow-growing A431 tumors have either no or very moderate sensitivity to DHODH inhibitors^37,38^. In accordance with these data, our results show that LFN treatment has a very limited effect on A431 and SCC-15 growth rate when used as a monotherapy.

Numerous cytokines, growth factors, and oncogenes activate STAT3 by affecting its post-translational modifications. Not surprisingly, therefore, over-activation of STAT3 and its pro-tumoral activity has been reported in a wide variety of tumors^39,40^. However, an increasing body of evidence supports the idea that STAT3 could have pro-tumorigenic or anti-tumorigenic activities depending on the specific tumor type, mutational landscape, stage of carcinogenesis, and metabolic conditions^41^. Therefore, targeting the downstream effectors of STAT3 with more narrow activities might increase the likelihood of developing an efficient anti-cancer drug. As a downstream effector, DHODH would be a promising target whose inhibition could be an effective anti-cancer strategy.

Altogether, our results suggest that DHODH can be targeted for both skin tumor prevention and curative combination therapy.

## Materials and methods

### Cell lines and culture

The origin and characteristics of the human cancer cell lines A431 and SCC-15 used in the present study are as follows. The A431 and SCC-15 lines, which were derived respectively, from the malignant epidermal carcinoma of an old female and old man, was obtained from the American Type Culture Collection (ATCC). Cells were grown in DMEM medium supplemented with 10% fetal calf serum (Gibco BRL Invitrogen, USA) and cultured in a humidified atmosphere of 5% CO_2_ at 37 °C. Both cell strains were cryopreserved within three passages and no cell aliquot was cultured continuously for more than 6 months. A431 and SCC-15 cell cultures were tested every 2 weeks for mycoplasma contamination by PCR and always came back negative. A431 and SCC-15 cell cultures were tested every 2 weeks for mycoplasma contamination by PCR and always came back negative. The cell lines were authenticated by short tandem repeat (STR) genotyping. The last authentication were performed just before transplantation of cells into mice. Cell lines used were 100% matched with those of ATCC and no cross-contamination of other human cells was observed.

### Animals and experimental protocol

SKH-1 hairless mice were purchased at 4–6 weeks of age from Charles River (L’arbresle, France). Mice were bred and maintained in a pathogen-free mouse facility. Female mice were used in these experiments. Mice were randomly assigned to each group before the start and experiments were performed blinded with respect to genotype and/or treatment. Experimental subgroups consisted of at least 10 mice per group. A UV Irradiation Unit (Daavlin, Bryan, OH) equipped with an electronic controller to regulate the dosage was used^42,43^. The UV dose was quantified with an X-96 dosimeter obtained from Daavlin. SKH-1 mice were exposed to UVB for 28 weeks (150 mJ/cm^2^, three times a week). Tumor numbers and volume were recorded every week. Tumor volume was estimated by measuring the shortest (width, ‘*W*’) and longest (length, ‘*L*’) axes, based on the volume of a cylinder with hemispherical ends, according to the following equation: calculated volume (mm^3^) = [*π*(*W*/2)^2^ (*L*−*W*)] + [4/3*π* (*W*/2)^3^].

For xenograft studies, NOD/Shi-SCID IL2Rγnull mice (NSG) were bred in standard conditions compliant with regulations and housed in a pathogen-free animal facility. Experimental subgroups consisted of 10 mice per group, with each subgroup caged separately. 5 × 10^5^ of A431 or SCC-15 cells combined with Matrigel^®^ Matrix High Concentration (Corning, USA) were subcutaneously (s.c.) injected in the right flank of mice. When the tumors reached 200–300 mm^3^ in volume, mice were randomly assigned to four groups. Treatments consisted of LFN alone (intraperitoneal injections of 20 mg/kg/day), 5-FU alone (intraperitoneal injections 20 mg/kg *t* times a week/day), or the combination of both drugs. Counterpart control groups received intraperitoneal injection of phosphate buffered saline (PBS). To assess the tumor volumes and growth rate of tumors, caliper measurements of the tumors were obtained every 3 days. Tumor volumes were calculated by the following formula: volume = (width)^2^ × length/2. After 18 days of treatment, mice were euthanized, and tumors were extracted and weighed.

All mouse experiments were carried out with the approval of Bordeaux University Animal Care and Use Committee and in accordance with relevant guidelines and regulations.

### Measurement of DHODH activity

The skin samples were put in a hypotonic buffer (2.5 mM Tris/HCl, pH 7.5 and 2.5 mM MgCl_2_) on ice for 15 min, homogenized and then sonicated for 15 s. The protein concentration for each sample was measured by BCA protein assay. DHO-ubiquinone oxidoreductase activity was measured spectrophotometrically (U-3210; Hitachi) at 37 °C by monitoring the decrease in absorbance at 600 nm of reduced DCPIP (2,6-dichlorophenol–indophenol; used as an artificial electron acceptor). Briefly, the reaction was initiated with 20 mM DHO in 1 ml of standard reaction buffer (containing 50 mM potassium phosphate, 5 mg/ml BSA and 2.5 mM MgCl_2_) supplemented with 50 μM DCPIP, 2 μg of rotenone (a complex I inhibitor), 2 μg of antimycin A (a complex III inhibitor), 5 mM NaN_3_ (a complex IV inhibitor) and 25 µg of skin lysate. The data were expressed as nmol min^−1^ μg^−1^ of protein. The reaction was stopped by the addition of 50 µM terileflunomide.

### RNA extraction and quantitative real-time RT-PCR (qRT-PCR)

Total epidermal RNA was extracted using TRIzol (Invitrogen) according to the manufacturer’s instructions. Total cellular RNA (2 μg) was reverse-transcribed at 42 °C for 60 min using the First Strand cDNA synthesis kit (Roche Applied Science). Quantitative real-time PCR was carried out for DHODH and tubulin using the SYBR Green method with a Bio-Rad instrument. Primer sequences used for PCR were as follows: *Dhodh*, forward primer (AGAGAGCTGGGCATCGAC) and reverse primer (AACCCCGATGATGGGAAT); *Tubulin* forward primer (CAAGGAGGATGCTGCCAATAA) and reverse primer (GCTGTGGAAAACCAAGAAGC). The reactions were cycled 40 times after initial polymerase activation (50 °C, 2 min) and initial denaturation (95 °C, 15 min) using the following parameters: denaturation at 95 °C for 15 s; and annealing and extension at 60 °C for 1 min. A final fusion cycle (95 °C, 30 s; 60 °C, 30 s; 95 °C, 30 s) terminated these reactions. The standard curve demonstrated a linear relationship between the Ct values and the cDNA concentration. The relative expression of each gene was assessed by considering the Ct and efficiency values and normalized according to the tubulin expression level.

### Determination of uridine concentration in plasma by HPLC

Blood samples from mice were collected in heparinized capillaries. The plasma was prepared by centrifugation. Perchloric acid (final concentration of 0.5 M) was added to the plasma followed by incubation on ice for 5 min prior to centrifugation (12,000 × *g*, 2 min at 4 °C). Supernatants were kept at −20 °C and directly used for HPLC analysis. The uridine was resolved on a Phenomenex Kinetex C_18_ (100 × 4.6 mm, 2.6 μm) column eluted with 100 mM ammonium acetate, pH 5.16 (solvent A) and acetonitrile (solvent B) with a linear gradient from 35% A (65%B) to 50% A (50%B) in 0.8 min with a flow rate of 0.35 ml/min. Uridine elutes at 0.51 min. Twenty microliters of standard and samples were injected. All standards were freshly prepared and dissolved in 35% 100 mM ammonium acetate/65% acetonitrile and frozen at −20 °C. The following standard calibration curve was used: 20, 50, 100, 200, 500, 1500, 2000, 5000, 10,000, 15,000 ng/mL.

### Quantification of CPDs by immunodot blot analysis

Mouse skin biopsies were incubated overnight at 65 °C in DirectPCR Lysis Reagent (Euromedex) and 2% proteinase K (Sigma). DNA was extracted by using sodium acetate/ethanol precipitation and quantified on a Nanodrop spectrophotometer. 500 ng of genomic DNA were mixed with 1% SYBR Green (*Brilliant III Ultra-Fast* SYBR^®^), dot-blotted onto a Hybond N+ nitrocellulose membrane (Amersham) and dried at 80 °C for 30 min. Membranes were blocked for 20 min (20 mM TBS, 5% non-fat dry milk, 0.5% Tween 20, pH 7.6) and incubated with anti-CPD monoclonal antibody (1:1000, Kamiya Biomedical) overnight at 4 °C. Membranes were washed in TBS and incubated for 1 h with an HRP-conjugated secondary antibody (1:2000, Vector Laboratories). Blots were developed using an ECL reagent (Biorad). Chemiluminescence signals were quantified and normalized against SYBR green fluorescence.

### Western blotting procedure

Western blotting was performed as previously described^44,45^. Briefly, equal amounts of total protein were resolved by SDS–polyacrylamide gel electrophoresis (SDS–PAGE) and electrophoretically transferred to PVDF membranes. The membranes were then incubated overnight at 4 °C with a 1:1000 dilution of the anti-DHODH (ab174288), anti-STAT3 (ab119352), anti-phosphorylated STAT3 (Abcam, Paris, France), and anti-β-actin (A2228, Sigma-Aldrich, Saint Quentin Fallavier, France) antibodies. After additional incubation with a 1:10,000 dilution of an anti-immunoglobulin horseradish peroxidase-linked antibody (Vector Laboratories, Biovalley S.A., Marne la Vallée, France) for 1 h, blots were developed using the chemiluminescence ECL reagent (Perkin Elmer, Courtaboeuf, France).

### Chromatin immunoprecipitation assay

The assay was performed using the EpiQuik^TM^ tissue Chromatin Immunoprecipitation Kit (Euromedex, Mundolsheim, France) in accordance with the manufacturer’s instructions. Briefly, to cross-link the proteins to DNA, skin specimens were incubated in medium containing 1% formaldehyde for 15 min at 37 °C. After adding glycine and lysis buffer, obtained chromatin lysate was sonicated to produce DNA of ~500 bp. Protein–DNA complexes were immunoprecipitated with anti-STAT3 antibody (ab119352, Abcam) or normal mouse IgG (negative control). The immunoprecipitated DNA was then purified and eluted with 50 µl of elution buffer. PCR amplification was done using 2 µl of DNA sample with different sets of primers (Supplementary Table S1). Amplification of soluble chromatin prior to immunoprecipitation was used as an input positive control. qRT-PCR was carried out on eluted DNA with the same primer sets, using the SYBR Green method with a Bio-Rad instrument. The reactions were cycled 40 times after initial polymerase activation (50 °C, 2 min) and initial denaturation (95 °C, 15 min) using the following parameters: denaturation at 95 °C for 15 s; and annealing and extension at 60 °C for 1 min. A final fusion cycle (95 °C, 30 s; 60 °C, 30 s; 95 °C, 30 s) terminated these reactions. The fold enrichment was calculated for each primer/antibody/treatment. The normalized ChIP Ct values were calculated: Δ*C*_t(normalized ChIP)_ = {*C*_t_(ChIP) − [*C*_t_(Input) − Log_2_ (input dilution factor)]}. The % Input was then calculated: % Input = 100 × 2^(−Δ*C*^_t_^(normalized ChIP)^. Finaly, fold enrichment was calculated using the following formula: fold enrichment = (% Input of antibody/%Input of IgG).

### Plasmid constructs and luciferase assays

A DHODH promoter reporter plasmid, in which 1.4 kb promoter region upstream from the ATG translation initiation codon of DHODH was cloned into pEZX-PG04 plasmid, was purchased from Tebu-bio (Le Perray-en-Yvelines, France). The mutations in GAS-2 to 5 were generated using a site-directed mutagenesis kit (Promega, Madison, WI, USA) according to the manufacturer’s instructions. The primers used to mutate the GAS-2 to 5 sites were as follows: GAS-2, 3: 5′-atgaggtaat***ttacaggGG***Gatgtatgggactggagatcatg***ttacatgGG***gcaagccaat-3′, GAS-4: 5′-aaaggactta***tttttagGG***tttatgtgta-3′, GAS-5: 5′-ggaagtatta***ttccagaGG***aggaagggga-3′, in which the GAS sequences are marked in bold italics and the mutated bases are marked in bold uppercase italics. The promoter regions containing 450 and 850 bp upstream from the ATG translation initiation codon of DHODH were amplified from this plasmid by PCR and cloned into pEZX-PG04 plasmid, which codes for a secreted Gaussia Luciferase (Gluc) as the promoter reporter and secreted alkaline phosphatase (SEAP) as the internal control for signal normalization. Keratinocytes isolated from non-irradiated and irradiated skin were co-transfected with siRNA-STAT3 (Santa Cruz technology) and 32 ng of promoter reporter plasmid. Medium were then collected 24 h after transfection, kept at −80 °C and then subjected to luciferase assays using the Secrete-Pair Dual Luminescence Assay Kit (Gluc, SEAP) (Tebu-Bio) in accordance with the manufacturer’s instructions. The Gluc activity expressed in each well was normalized by SEAP. For all transfection assays, at least three independent experiments were performed in triplicate.

### Statistics

For all groups that were statistically compared, the variance between the groups was very similar. Comparisons between two groups were calculated using Student’s *t*-test (two tailed) and a *P-*value < 0.05 (*) was considered significant. Results are presented as means ± SD. Comparisons between more than two groups were calculated with one-way analysis of variance (ANOVA) followed by Bonferroni’s multiple comparison tests. To assess the number and the volume of tumors, a two-way ANOVA analysis of variance test followed by a post hoc Bonferroni’s test was used. A *P*-value < 0.05 (*) was considered significant. Results are presented as means ± SEM.

## Supplementary information

Supplementary information

## Data Availability

The authors declare that the data supporting the findings of this study are available within the paper and its Supplementary Information files. Additional data are available from the corresponding author upon reasonable request.

## References

[1] Rogers HW, Weinstock MA, Feldman SR, Coldiron BM (2015). Incidence estimate of nonmelanoma skin cancer (keratinocyte carcinomas) in the US population, 2012. JAMA Dermatology.

[2] Donaldson MR, Coldiron BM (2011). No end in sight: the skin cancer epidemic continues. Semin. Cutan. Med. Surg..

[3] Ratushny V, Gober MD, Hick R, Ridky TW, Seykora JT (2012). From keratinocyte to cancer: the pathogenesis and modeling of cutaneous squamous cell carcinoma. J. Clin. Invest..

[4] Karia PS, Han J, Schmults CD (2013). Cutaneous squamous cell carcinoma: estimated incidence of disease, nodal metastasis, and deaths from disease in the United States, 2012. J. Am. Acad. Dermatol..

[5] Cranmer LD, Engelhardt C, Morgan SS (2010). Treatment of unresectable and metastatic cutaneous squamous cell carcinoma. Oncologist.

[6] Stratigos A (2015). Diagnosis and treatment of invasive squamous cell carcinoma of the skin: European consensus-based interdisciplinary guideline. Eur. J. Cancer.

[7] Que SKT, Zwald FO, Schmults CD (2018). Cutaneous squamous cell carcinoma: management of advanced and high-stage tumors. J. Am. Acad. Dermatol..

[8] Martinez JC, Otley CC, Okuno SH, Foote RL, Kasperbauer JL (2004). Chemotherapy in the management of advanced cutaneous squamous cell carcinoma in organ transplant recipients: theoretical and practical considerations. Dermatol. Surg..

[9] Migden MR (2018). PD-1 blockade with cemiplimab in advanced cutaneous squamous-cell carcinoma. N. Engl. J. Med..

[10] Kaplan MJ (2001). Leflunomide Aventis Pharma. Curr. Opin. Investig. Drugs.

[11] McLean JE, Neidhardt EA, Grossman TH, Hedstrom L (2001). Multiple inhibitor analysis of the brequinar and leflunomide binding sites on human dihydroorotate dehydrogenase. Biochemistry.

[12] Schiff MH, Strand V, Oed C, Loew-Friedrich I (2000). Leflunomide: efficacy and safety in clinical trials for the treatment of rheumatoid arthritis. Drugs Today.

[13] Hosseini M (2018). Energy metabolism rewiring precedes UVB-induced primary skin tumor formation. Cell Rep..

[14] Hosseini M, Ezzedine K, Taieb A, Rezvani HR (2015). Oxidative and energy metabolism as potential clues for clinical heterogeneity in nucleotide excision repair disorders. J. Invest. Dermatol.

[15] Vallania F (2009). Genome-wide discovery of functional transcription factor binding sites by comparative genomics: the case of Stat3. Proc. Natl Acad. Sci. USA.

[16] Kim DJ, Angel JM, Sano S, DiGiovanni J (2009). Constitutive activation and targeted disruption of signal transducer and activator of transcription 3 (Stat3) in mouse epidermis reveal its critical role in UVB-induced skin carcinogenesis. Oncogene.

[17] Galluzzi L, Kepp O, Vander Heiden MG, Kroemer G (2013). Metabolic targets for cancer therapy. Nat. Rev. Drug Discov..

[18] Smolkova K (2011). Waves of gene regulation suppress and then restore oxidative phosphorylation in cancer cells. Int. J. Biochem. Cell Biol..

[19] Ward PS, Thompson CB (2012). Metabolic reprogramming: a cancer hallmark even warburg did not anticipate. Cancer Cell.

[20] Brown KK, Spinelli JB, Asara JM, Toker A (2017). Adaptive reprogramming of de novo pyrimidine synthesis is a metabolic vulnerability in triple-negative breast cancer. Cancer Discov..

[21] Hanson K (2018). The anti-rheumatic drug, leflunomide, synergizes with MEK inhibition to suppress melanoma growth. Oncotarget.

[22] Fitzpatrick LR (2010). 4SC-101, a novel immunosuppressive drug, inhibits IL-17 and attenuates colitis in two murine models of inflammatory bowel disease. Inflamm. Bowel Dis..

[23] Fox RI (1998). Mechanism of action of leflunomide in rheumatoid arthritis. J. Rheumatol. Suppl..

[24] He D (2012). Teriflunomide for multiple sclerosis. Cochrane Database Syst. Rev..

[25] Leban J, Saeb W, Garcia G, Baumgartner R, Kramer B (2004). Discovery of a novel series of DHODH inhibitors by a docking procedure and QSAR refinement. Bioorg. Med. Chem. Lett..

[26] Braakhuis BJ, van Dongen GA, Peters GJ, van Walsum M, Snow GB (1990). Antitumor activity of brequinar sodium (Dup-785) against human head and neck squamous cell carcinoma xenografts. Cancer Lett..

[27] Chen SF, Perrella FW, Behrens DL, Papp LM (1992). Inhibition of dihydroorotate dehydrogenase activity by brequinar sodium. Cancer Res..

[28] Dexter DL (1985). Activity of a novel 4-quinolinecarboxylic acid, NSC 368390 [6-fluoro-2-(2’-fluoro-1,1’-biphenyl-4-yl)-3-methyl-4-quinolinecarb oxylic acid sodium salt], against experimental tumors. Cancer Res..

[29] Strawn LM (2000). Effects of SU101 in combination with cytotoxic agents on the growth of subcutaneous tumor xenografts. Clin. Cancer Res..

[30] Bajzikova M (2019). Reactivation of dihydroorotate dehydrogenase-driven pyrimidine biosynthesis restores tumor growth of respiration-deficient cancer cells. Cell Metab..

[31] Arteaga CL (1989). Phase I clinical and pharmacokinetic trial of Brequinar sodium (DuP 785; NSC 368390). Cancer Res..

[32] Peters GJ (1990). In vivo inhibition of the pyrimidine de novo enzyme dihydroorotic acid dehydrogenase by brequinar sodium (DUP-785; NSC 368390) in mice and patients. Cancer Res..

[33] Schwartsmann G (1989). Pharmacokinetics of Brequinar sodium (NSC 368390) in patients with solid tumors during a phase I study. Eur. J. Cancer Clin. Oncol..

[34] Sykes DB (2016). Inhibition of dihydroorotate dehydrogenase overcomes differentiation blockade in acute myeloid leukemia. Cell.

[35] Mohamad Fairus AK, Choudhary B, Hosahalli S, Kavitha N, Shatrah O (2017). Dihydroorotate dehydrogenase (DHODH) inhibitors affect ATP depletion, endogenous ROS and mediate S-phase arrest in breast cancer cells. Biochimie.

[36] Khutornenko AA (2010). Pyrimidine biosynthesis links mitochondrial respiration to the p53 pathway. Proc. Natl Acad. Sci. USA.

[37] Shawver LK (1997). Inhibition of platelet-derived growth factor-mediated signal transduction and tumor growth by N-[4-(trifluoromethyl)-phenyl]5-methylisoxazole-4-carboxamide. Clin. Cancer Res..

[38] Xu X (1999). In vitro and in vivo antitumor activity of a novel immunomodulatory drug, leflunomide: mechanisms of action. Biochem. Pharmacol..

[39] Yu H, Lee H, Herrmann A, Buettner R, Jove R (2014). Revisiting STAT3 signalling in cancer: new and unexpected biological functions. Nat. Rev. Cancer.

[40] Yuan J, Zhang F, Niu R (2015). Multiple regulation pathways and pivotal biological functions of STAT3 in cancer. Sci. Rep..

[41] Avalle L, Camporeale A, Camperi A, Poli V (2017). STAT3 in cancer: a double edged sword. Cytokine.

[42] Mahfouf W (2019). Loss of epidermal HIF-1alpha blocks UVB-induced tumorigenesis by affecting DNA repair capacity and oxidative stress. J. Invest. Dermatol..

[43] Raad H (2017). NADPH oxidase-1 plays a key role in keratinocyte responses to UV radiation and UVB-induced skin carcinogenesis. J. Invest. Dermatol..

[44] Rezvani HR (2011). XPC silencing in normal human keratinocytes triggers metabolic alterations that drive the formation of squamous cell carcinomas. J. Clin. Invest.

[45] Rezvani HR (2011). XPC silencing in normal human keratinocytes triggers metabolic alterations through NOX-1 activation-mediated reactive oxygen species. Biochim. Biophys. Acta.

