# Supplementary material for: UVB-induced DHODH upregulation, which is driven by STAT3, is a promising target for chemoprevention and combination therapy of photocarcinogenesis
Source: Oncogenesis. 2019 Sep 24;8(10):52. doi: 10.1038/s41389-019-0161-z (PMC6760220; doi:10.1038/s41389-019-0161-z)
Supplement: Supplementary file 1 — Supplementary information [file 41389_2019_161_MOESM1_ESM.docx]

**Supplementary Information**

**Supplementary Table S1. Primer sequences for ChIP assays**

| Target sequence | sequence | Amplified region |
| --- | --- | --- |
| GAS-1 | F: TTCAATCACTAACCCAAACTCC  R: TCTGTTTGTCTGTTACTGCTTCC | -1469/-1317 |
| GAS-2.3.4 | F: TGGAACAACATTCAACCATTAGA  R: GGTTTTCCCATACCAACAGG | -1282/-1077 |
| GAS-5 | F: CTCCAGCCTCCTGTAACCTG  R: AGAGGAGGGCATTGAATCCT | -949/-773 |
| GAS-6.7 | F: AGAGGCACTTGAGTGCTGAG  R: TGCAAACACACAAAAATTTACC | -750/-605 |
| GAS-8 | F: CCACCACTGCCCTGATAAAA  R: AAGGAAACAAAGGGCGATG | -448/-318 |
